# Early Life Factors and Type 2 Diabetes Mellitus

**DOI:** 10.1155/2013/485082

**Published:** 2013-12-16

**Authors:** Xinli Jiang, Huijie Ma, Yan Wang, Yan Liu

**Affiliations:** ^1^Department of Ophthalmology, The Third Hospital of Hebei Medical University, Ziqiang Road 139, Shijiazhuang, Hebei 050051, China; ^2^Department of Physiology, Hebei Medical University, Zhongshan Road 361, Shijiazhuang, Hebei 050017, China; ^3^Department of Endocrinology, The Third Hospital of Hebei Medical University, Ziqiang Road 139, Shijiazhuang, Hebei 050051, China; ^4^Orthopaedic Biomechanical Laboratory of Hebei Province, The Third Hospital of Hebei Medical University, Ziqiang Road 139, Shijiazhuang, Hebei 050051, China

## Abstract

Type 2 diabetes mellitus (T2DM) is a multifactorial disease, and its aetiology involves a complex interplay between genetic, epigenetic, and environmental factors. In recent years, evidences from both human and animal experiments have correlated early life factors with programming diabetes risk in adult life. Fetal and neonatal period is crucial for organ development. Many maternal factors during pregnancy may increase the risk of diabetes of offsprings in later life, which include malnutrition, healthy (hyperglycemia and obesity), behavior (smoking, drinking, and junk food diet), hormone administration, and even stress. In neonates, catch-up growth, lactation, glucocorticoids administration, and stress have all been found to increase the risk of insulin resistance or T2DM. Unfavorable environments (socioeconomic situation and famine) or obesity also has long-term negative effects on children by causing increased susceptibility to T2DM in adults. We also address the potential mechanisms that may underlie the developmental programming of T2DM. Therefore, it might be possible to prevent or delay the risk for T2DM by improving pre- and/or postnatal factors.

## 1. Introduction

Type 2 diabetes (T2DM) is a metabolic disease caused by genetic and multiple environmental factors. Epidemical and experimental studies have found that detrimental early life factors may predispose high incidence of cardiovascular disease and metabolic diseases in later life, which is also termed as “barker hypothesis.” Organs are under development and functional maturation from fetal stage to childhood; disturbance of the homeostasis during crucial periods might predispose increased risk of insulin resistance and even T2DM in late life.

## 2. Part I: Prenatal Factors (Figure 1)

### 2.1. Diet and Nutrition

It has been suggested that the quality and quantity of the nutrition during pregnancy may cause strong and permanent effects on the fetus. The altered structure of chromosome during this procedure might be the cause of cell dysfunction and increased susceptibility to diseases through altered gene expression [1].

#### 2.1.1. Malnutrition and Low Protein Diet

The associations between maternal malnutrition, low protein diet, and T2DM have been widely studied. Typical epidemical studies from the population born during the Dutch famine period [2] or in some poor countries [3] have found that those who had been exposed to maternal malnutrition may have increased morbidity of metabolic diseases including T2DM in adult life.

The mechanisms responsible for the prenatal malnutrition programming insulin resistance or T2DM remain unclear. Orozco-Solís et al. [4] have found that low protein diet during pregnancy and lactating may cause permanent altered hypothalamic expression of genes in rat offspring involved in insulin signaling and lipid and glucose metabolism, which may programme metabolic diseases.

**Figure 2:**
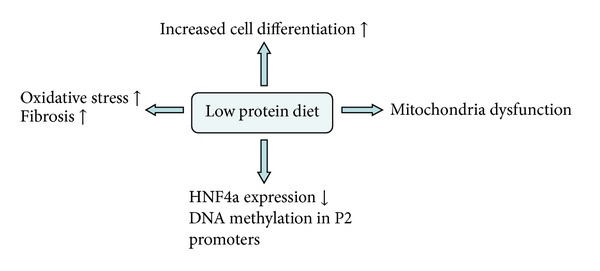
The effect of low protein diet during pregnancy on postnatal *β* cell. Low protein diet during pregnancy may lead to increased oxidative stress, fibrosis, decreased HNF4a expression, defected mitochondriogenesis, and mitochondria dysfunction, and increased cell differentiation instead of proliferation was found in *β* cell of adult animal offspring, which may participate in *β*-cell dysfunction and consequently increase the incidence of T2DM.

In addition, the effect of low protein diet during pregnancy on postnatal *β* cell has also been noticed recently. Increased oxidative stress and fibrosis [5], decreased HNF4a expression with increased DNA methylation in P2 promoters [6], defected mitochondriogenesis and mitochondria dysfunction [7], and increased cell differentiation instead of proliferation [8] were found in *β* cell of adult animal offspring whose mothers were under low protein diet during pregnancy. These may cause *β*-cell dysfunction and consequently increase the incidence of T2DM in postnatal life (Figure 2).

#### 2.1.2. Overnutrition

*High Protein.* A study from Maurer and Reimer [9] in Wistar rats has found that high protein diet during pregnancy and lactating may cause increased resistin and IL-6 mRNA levels in brown fat tissue in 35-day-old offspring; both factors were included in the pathogenesis of insulin resistance [10, 11].

*High Fat Diet.* Both human and animal studies have identified that fat diet may cause obesity and insulin resistance [12, 13]. Intriguingly, the effect of high fat diet on metabolic disorders seemed to be programmative. The prenatal period is a key developmental window for nutrition status. Masuyama and Hiramatsu [14] found that mice offspring exposed to high fat diet during pregnancy developed insulin resistance and hyperlipidemia at 24 wks of age, which was associated with altered levels of leptin in adipose tissue. The experiment conducted in C57BL/6 mice by Liang et al. [15] has also showed that high saturated fatty acids diet during pregnancy led to insulin resistance, hyperglycemia in adult offspring under normal diet condition. The mechanisms underlying are still under investigation. Evidences from animal study have indicated that overexposure to high fat diet in utero may lead to elevated mRNA level of hypothalamic signal transducer and activator of transcription-3 and suppressor of cytokine signalling-3 in the offspring [16]. Both of these two factors are found to participate in obese and insulin resistance cases [17]. In addition, prenatal exposure to high saturated fats may cause increased hepatic phosphoenolpyruvate carboxykinase expression, fatty liver, reduced basal acetyl CoA carboxylase phosphorylation, and insulin signalling [18]. Impaired Wnt/*β*-catenin signaling pathway in skeletal muscle has also been found [19], which may also participate in pathogenesis of insulin resistance in adult life, since the insulin sensitivity can be improved by activating Wnt/*β*-catenin [20].

#### 2.1.3. Transfatty Acids and Junk Food

Transfatty acids are unsaturated fatty acids that contain nonconjugated double bond in the trans-configuration. So far, data that correlated transfatty acids diet with insulin resistance or diabetes is weak and inconsistent [21–23]. However, it is still worth noting that prenatal exposure to transfatty acids might cause impaired insulin resistance and increased content of abdominal fat after birth [24]. No similar effects can be observed in mice exposed to transfatty acid during lactating.

Data about long-term effects of prenatal junk food taking is quite limited; experiment from Bayol et al. [25] has indicated that junk food taking during prenatal and lactating period may cause reduced insulin sensitivity in female offspring rats. More intensive studies still need to be performed for the convincing conclusion.

#### 2.1.4. Alcohol

Studies have already correlated chronic alcohol intake with insulin resistance even T2DM [26, 27]. In the series of studies performed by Chen and Nyomba they have found that SD rats with alcohol intake (4 g/kg/day) during pregnancy may have hyperglycemia and reduced glucose transporter type 4 (GLUT4) content in muscle in adult offspring after a reduced birth weight and then catch up growth [28]. In addition, in this animal model, impaired inhibition effects of insulin on hepatic gene expression of phosphoenolpyruvate carboxykinase and peroxisome proliferator activated receptor gamma coactivator-1 mRNA [29] and reduced phosphorylation of protein kinase C zeta isoform [30] were exhibited in the offspring. Yao et al. [31] have found prenatal alcohol intake elevated expression of Tribbles 3 and phosphatase and tensin homolog deleted on chromosome 10 in both liver and muscle [32, 33], leading to impaired insulin sensitivity [34, 35]. In addition, increased 11beta-hydroxysteroid dehydrogenase type-1 level in liver and adipose tissue [36] may also partly contribute to the insulin resistance caused by prenatal alcohol taking though elevating local glucocorticoid levels.

### 2.2. Environmental Factors

#### 2.2.1. Biophenol A

Biophenol A, a biochemical material used in plastic containers that are widely used in daily life [37], has been found that it may achieve similar effects with estrogen [38, 39]. Studies have supported that biophenol A might be correlated with the pathogenesis of T2DM [40, 41]. A human study performed by Lang et al. [42] has shown that the biophenol A concentration in urine positively correlated with cardiovascular diseases and diabetes.

In rats, 50 *μ*g/kg·d biophenol A intake during pregnancy and lactating period may lead to insulin resistance in adult offspring, and this effect can be largely enhanced by high fat diet after birth [43]. Similar results have also been described by Alonso-Magdalena et al. [44] who further found that the altered Ca2+ signaling pathway and reduced cell numbers in pancreas might contribute to reduced insulin sensitivity. However, controversial conclusions have also been raised out by Ryan et al. [45] in CD-1 mice, which indicate that perinatal exposure to ecologically relevant dose of BPA could not impair the glucose tolerance in the offspring. Therefore, different biophenol A dosages applied in different animal models may vary the conclusion.

#### 2.2.2. Maternal Hypoxia

Data from animal experiment has found that exposure to hypoxia during pregnancy leads to insulin resistance, impaired glucose homeostasis, and altered expression of genes involved in insulin-signaling pathways in the offspring [46]. Mechanisms underlying this relationship are unclear since intrauterine hypoxia may partly correlate with undernutrition. However, Camm et al. [47] found that, compared to prenatal undernutrition, prenatal hypoxia may cause different gene expression patterns in the liver and muscle in adult offspring, including reduced expression of hepatic insulin receptor substrate 1, phospho-Akt, and muscle Akt2, indicating that prenatal hypoxia may promote markers of insulin resistance independent of undernutrition.

#### 2.2.3. Maternal Smoking

Studies have reported the unfavorable effects of smoking on diabetes in adult [48, 49]. However, a clearly causal relationship has only been found between maternal smoking and increased risk of T2DM in the offspring. A human study performed by Thiering et al. [50] had found increased insulin levels in 10-year-old children after prenatal smoking, and breast milk feeding made this alteration even more magnificent. This finding is consistent with the study performed previously by Bruin et al. [51] in animals which indicated that both conception and lactation periods were needed for nicotine exposure that may result in permanent *β*-cell loss and subsequent impaired glucose tolerance. In addition, Holloway et al. [52] found that fetal and neonatal exposure to nicotine has transgenerational effects and insulin resistance can be found in the F2 offspring. Exact mechanism still remains largely unknown; reduced pancreas cell numbers and size and reduced expression of *β*-cell marker genes such as pdx-1, Pax-1, and Nkx6.1 [53] all have been addressed. In addition, data from Chen et al. have indicated that nicotine may also downregulate gene expression of appetite regulators neuropeptide Y and pro-opiomelanocortin in the arcuate nucleus of the hypothalamus in fetal brain, which may consequently lead to unhealthy eating habits in the offspring and predispose high risk of obesity or diabetes [54].

### 2.3. Prenatal Psychological Stress

It is already known that exposure to high levels of maternal stress hormones during pregnancy may produce detrimental effects on the offspring [55]. The effect of prenatal stress in programming T2DM has been found in both human and animal studies [56–58]. A retrospective study has shown that children exposed to stress caused by bereavement during their prenatal life had more risk to T2DM later in life [57]. Another human data from Entringer et al. [58] found that maternal stressful life experiences may cause significantly elevated 2-hour insulin and C-peptide levels under glucose tolerance test in young adult offspring, indicating insulin resistance, which is independent of birth weight and family history of diabetes. The elucidation of the mechanism underlying this relationship is still not clear. A finding from a human study has shown that prenatal stress leads to shorter leukocyte telomere length in adult offspring [59], which has also been found to positively correlate with the pathogenesis diabetes [60] and children obese [61].

### 2.4. The Metabolic Situation during Pregnancy

#### 2.4.1. Obesity

Maternal obesity has risen dramatically over the past 20 years. Evidences from human and animal studies suggest that maternal obesity in pregnancy predisposes hyperinsulinemia, insulin resistance, and T2DM in the offspring [62–65]. Shankar et al. [66] have found in mice that the male offspring with overweight mother may exhibit magnificent increase in body weight and adipose tissue content, which also combined with insulin resistance and increased levels of insulin, leptin, and resistin. The precise underlying mechanisms that contribute to increased susceptibility of offspring to develop insulin resistance in later life remain poorly understood. Both increased number of apoptosis of the fetal pancreas *β* cell [67] and accelerated fetal *β*-cell growth and cell proliferation (which was regarded as overload working and consequently end up to *β* cell failure) [68] were observed in animal offsprings with obese mother, which may all contribute to the increased blood glucose level after birth. In addition, increased hepatic lipogenesis and fatty liver disease [69, 70] found in the offspring exposed to maternal obesity also contribute to hepatic insulin resistance.

#### 2.4.2. High Gestational Glucose Concentration

Exposure to elevated intrauterine glucose environment has been found to cause alterations in fetal growth patterns, which predispose these infants to developing obesity, insulin resistance, and diabetes later in life. So far the effects of intrauterine hyperglycemia on the offspring have been studied in human in pregnant mothers with T2DM or with gestational diabetes and in diabetic animal models mainly caused by streptozotocin treatment. Data accumulated from theses studies uniformly show glucose intolerance in the offspring. Human study performed by Boerschmann et al. [71] has indicated that, compared with those children with T1DM and normal glycemia mothers, children with mothers with gestational diabetes mellitus exhibit overweight and increased HOMA-IR. Another study from Bush et al. [72] in 5–10-year-old children also found that maternal gestational glucose concentration was inversely associated with offspring insulin sensitivity. Insulin resistance was also observed in rodent offspring prenatally under hyperglycemia environment caused by streptozotocin injection [73, 74]. It seems that in utero “diabetic” environment in which the fetus develops can increase the risk of diabetes in the child. In addition to genetic susceptibility, blunted insulin sensitivity in the offspring might largely contribute to this correlation. Relative gene expression was only explored in animal models which indicated that intrauterine hyperglycemia induced by streptozotocin injection resulted in increased hepatic gluconeogenic gene expression of glucose-6-phosphatase and phosphoenolpyruvate carboxykinase in the offspring [74] and the adult offspring of this cohort are prone to develop insulin resistance under high fat diet [73]. A human study found that maternal diabetes might cause an inherent defect in *β*-cell glucose sensitivity in the adult offspring [75].

### 2.5. Maternal Hormone Levels during Pregnancy

#### 2.5.1. Prenatal Testosterone

Prenatal testosterone overexposure has been considered to be correlated with polycystic ovary syndrome in adult female and was widely studied [76, 77]. Animal experiments performed in sheep [78, 79], rodents [80–82], and even monkeys [83] have all confirmed that prenatal testosterone overexposure leads to insulin resistance in the offspring. Testosterone overexposure during fetal development may impair insulin sensitivity pathways in both liver and muscle [79], increase hepatic gluconeogenesis [84], and impair pancreas islet response to glucose [80] in the offspring.

#### 2.5.2. Prenatal Glucocorticoids

Synthetic glucocorticoids have been used in pregnant women who are at risk of preterm delivery to promote fetal lung maturation. However, concerns have already emerged about the metabolic disorders caused by prenatal glucocorticoids excess. Studies from animal models have found that prenatal glucocorticoids treatment leads to increased hepatic gene expression of hepatocyte nuclear factor 4 alpha [85], phosphoenolpyruvate carboxykinase [86], and glucose-6-phosphatase [87] in the offspring, indicating elevated hepatic gluconeogenesis and hepatic insulin resistance. Nyirenda et al. also found that prenatal dexamethasone administration during late gestation may result in elevated 11 beta-HSD1 [88] and glucocorticoids receptor [86] expression in the liver, which may cause insulin resistance by increasing local glucocorticoids level [89] or activity.

Similar phenomenon has also been found when increased endogenous glucocorticoids pass through maternal to fetus. So far, maternal nicotine [90], food or energy restriction [91], and alcohol intake [92] have all been found to impair placental barrier and consequently cause increased endogenous glucocorticoids in utero.

## 3. Part II: Postnatal Factors

### 3.1. New Born 

#### 3.1.1. Catch-Up Growth

Catch-up growth, which appeared after lower birth body weight, is the issue that has been studied for years. Accumulated data suggest that low birth weight and catch-up growth are strongly associated with increased risk of insulin resistance and type 2 diabetes [93–96]. Intriguingly, different periods of catch-up growth seem to cause different effects on glucose tolerance and insulin sensitivity. Catch-up growth only in the first year after birth seems to have no effect on insulin sensitivity in 7-year-old child [97], while sustained catch-up growth (more than 1 year after birth) leads to higher insulin levels in 7-year-old child [97] or insulin resistance in 8-year-old child [98]. Compelling evidences raise the thrifty “catch-up fat” mechanisms, indicating that this growth trajectory is characterized by a disproportionately higher rate of fat gain and redistribution of glucose from skeletal muscle to adipose tissue, contributing to insulin resistance in skeletal muscle while hyperresponsiveness to insulin in adipose tissue [99–101].

#### 3.1.2. Lactation

**Figure 3:**
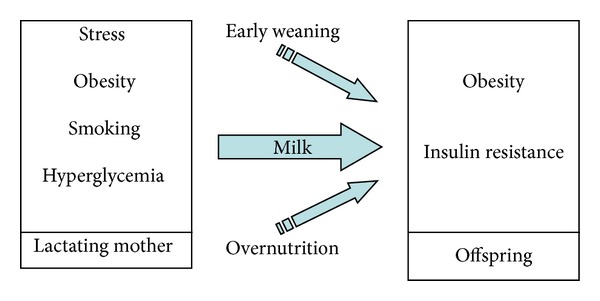
Lactation and insulin resistance. It has been found that both early weaning and overfeeding by more milk intake may lead to insulin resistance in later life. Maternal stress, obesity, hyperglycemia, and even smoking during lactation might also cause reduced insulin sensitivity in the offspring, which suggest that the breast milk can be the “agent,” transferring altered levels of hormones, insulin, or fatty acid contents from maternal circulation to neonate.

Lactation is also a sensitive period for the programming of later metabolic disorders (Figure 3).

*Early Weaning*. Early weaning may lead to undernutrition, which consequently program metabolic disorders later in life. Hyperglycaemia, higher insulin resistance index, and hyperleptinemia were observed in 6-month-old rats that were weaned early, which were accompanied with central leptin resistance [102].

*Overnutrition.* Overnutrition during lactation period is associated with metabolic disorders in later life. An experiment conducted in male mice by Pentinat et al. [103] has found that overgrowth mice caused by reduced pups per dams during lactation may develop metabolic disorders at the age of 4 months, including obesity, insulin resistance, and glucose intolerance. Similar results have also been found by Plagemann et al. in their serial experiments performed on rats [104, 105]. Strikingly, the effect of neonatal overnutrition on diabetes risk can be “inherent” to subsequent generations. Impaired glucose tolerance was found in the adult male mice offspring with the father overfed neonatally, and peripheral insulin resistance was found in the grand offspring, although these two generations of animals were not exposed to overnutrition during the neonatal time [103]. Increased oxidative stress in liver and reduced hepatic insulin signaling pathways [106] may underlie effects of neonatal overfeeding. Moreover, early overfeeding leads to permanent dysregulation of hypothalamic circuits in animal models, including reduced negative feedback to the satiety signal insulin on medial arcuate neurons in juvenile as well as adult rats [104] and increased hypothalamic insulin receptor promoter methylation ratio [105]. This may lead to functional resistance to insulin and leptin, which may underlie permanently an increase in food intake, overweight, and insulin resistance.

*Maternal Situation during Lactation.* Maternal physical or pathological situation during lactation may imprint elevated risk of metabolic diseases in the offspring. Experiments in animals have showed that mother under stress [107], with obesity [108], and exposed to nicotine [109] during lactating period may lead to obesity and insulin resistance in adult offspring, which implicates that the postnatal maternal environment is a major effecter of metabolic outcome in the offspring. It is also found that fostering nondiabetic offspring to diabetic dams may produce smaller offspring with altered arcuate nucleus neuropeptide Y, agouti-related peptide, and pro-opiomelanocortin expression [108]. The underlying mechanism is still far more conclusive. Therefore, the breast milk can be the “agent”; altered levels of hormones, insulin, or fatty acid contents may enter the milk from maternal circulation and then can be transferred to neonate.

#### 3.1.3. Neonatal Stress

Limited data from animal studies have found that stress caused by handling during the neonatal period may also be detrimental. Studies have found that neonatal mice, which were under maternal separation plus subcutaneous sham injection during the lactation period, developed hyperglycemia, hyperinsulinemia, hyperleptinemia, and hyperlipidemia in adult under fasting [110, 111]. Increased plasma corticosterone and adrenocorticotropin were found in these animals [110, 111] which might be responsible for the “diabetic” alteration.

#### 3.1.4. Neonatal Hormone Exposure

There are evidences indicated that exposure to some hormones during neonatal life may predispose metabolic disorders in adult life. Glucocorticoids treatment in neonatal rats caused increased fasting and postprandial blood glucose, which is combined with magnificent insulin resistance and lipid disorder in later life [112]. In addition, in newborn female rats, one subcutaneous injection with 0.35 mg oestradiol benzoate led to reduced insulin sensitivity in adult life by inducing inflammation and disturbance glucose metabolism in skeletal muscle [113], while 1 mg testosterone injection to female neonatal rats caused insulin resistance and increased mesenteric adipose tissue content in adult life [114].

#### 3.1.5. Neonatal Monosodium Glutamate Intake

Monosodium glutamate (MSG) is the sodium salt of glutamic acid, and it is a flavor enhancer that is widely used in Chinese food. The study of neonatal MSG treatment on neonatal animals has been performed since the 1970s and so far there are plenty of animal experiments that have evidenced detrimental effects of MSG administration during early life time, including growth retardation, retinal degeneration, and increased proinflammation in hippocampus [115–117]. In 1997, Hirata et al. found that MSG-treated animals developed central obesity, altered glucose tolerance, and hyperinsulinaemia [118]. More similar evidences have been documented in later experiments [119–121], indicating that neonatal MSG treatment may lead to increased risk of diabetes in adult life. MSG may cause obesity and nonfatty liver [120, 122] and increased mRNA level of IL-6, TNF*α*, resistin, and leptin in visceral fat tissue [119], which might all predispose insulin resistance in later life.

### 3.2. Childhood

#### 3.2.1. Low Socioeconomic Status

Socioeconomic status has a notable impact on health disparities, including type 2 diabetes risk. Low childhood socioeconomic status was found linked to type 2 diabetes in some studies [123, 124] and the association remained even after being adjusted for adult socioeconomic status and obesity. Low childhood socioeconomic status was considered to be a robust independent factor of incidence of type 2 diabetes in adulthood and the risk was found greater when childhood socioeconomic status combined with adult obesity. Poor nutrition, unhealthy behaviors, and limited access to material goods and limited socioeconomic opportunities may contribute to altered body composition in later life, which might explain the relationship between childhood socioeconomic position and metabolic disorders in adult.

#### 3.2.2. Famine

Undernutrition during childhood has been found to be associated with an increased type 2 diabetes risk in adulthood. Study in women who had experienced Dutch famine has shown that short period of moderate or severe undernutrition during childhood increases type 2 diabetes risk in adulthood [125].

#### 3.2.3. Obesity

Childhood obesity is an issue of serious medical and social concern. Many studies have demonstrated the positive correlations between childhood obesity and adult metabolic disorders, including type 2 diabetes [61, 126, 127]. Obesity, which mostly caused by high caloric food intake, may always combine with insulin resistance [128]. An unfavorable programming of body composition could be one mechanism linking early childhood growth with later increased risk for type 2 diabetes. In addition, a study performed in 793 French children aged 2–17 yr has suggested that obese children have significantly shorter leukocyte telomeres than their nonobese counterparts [61]. Leukocyte telomere length (LTL), a marker of biological age, is associated with age-related conditions including cardiovascular disease and type 2 diabetes which highlights a potentially deleterious impact of early onset obesity on future health.

## 4. Conclusion

There is increasing recognition that the risk of type 2 diabetes can be influenced by prenatal, neonatal, and childhood exposures. In the present studies, we have reviewed nutritional, environmental, and physiological factors from prenatal to postnatal periods, which have been documented in studies that may correlate with insulin resistance or type 2 diabetes in adult life. Further investigations are still required. However, relative knowledge education might be successful in women of child-bearing age and ultimate to reduce the disease risk in their potential offspring.

## Figures and Tables

**Figure 1:**
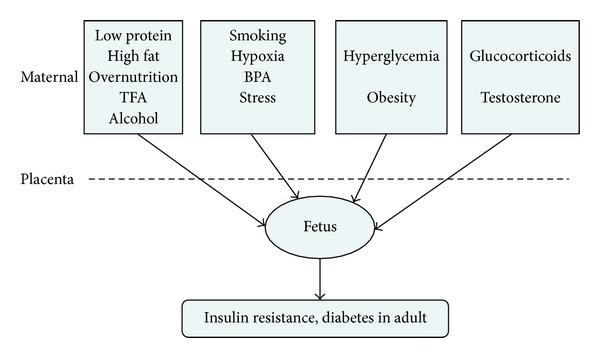
Prenatal factors mentioned in recent years that might correlate with insulin resistance and/or T2DM. Data from human and animal studies have shown that malnutrition or overnutrition, metabolic disorders, exposure to hypoxia, some chemicals and hormones, and unhealthy lifestyle such as smoking and alcohol drinking during pregnancy might predispose detrimental long-term effects on offspring, leading to increased risk of insulin resistance or T2DM. TFA: transfatty acids; BPA: biophenol A.
